# Dysregulation of the IFN-γ-STAT1 signaling pathway in a cell line model of large granular lymphocyte leukemia

**DOI:** 10.1371/journal.pone.0193429

**Published:** 2018-02-23

**Authors:** Paige M. Kulling, Kristine C. Olson, Cait E. Hamele, Mariella F. Toro, Su-Fern Tan, David J. Feith, Thomas P. Loughran

**Affiliations:** 1 University of Virginia Cancer Center, University of Virginia; Charlottesville, VA United States of America; 2 Department of Medicine, Division of Hematology/Oncology, University of Virginia; Charlottesville, VA United States of America; 3 Department of Pathology, University of Virginia; Charlottesville, VA United States of America; Institut Curie, FRANCE

## Abstract

T cell large granular lymphocyte leukemia (T-LGLL) is a rare incurable disease that is characterized by defective apoptosis of cytotoxic CD8+ T cells. Chronic activation of the Janus Kinase-Signal Transducer and Activator of Transcription (JAK-STAT) pathway is a hallmark of T-LGLL. One manifestation is the constitutive phosphorylation of tyrosine 701 of STAT1 (p-STAT1). T-LGLL patients also exhibit elevated serum levels of the STAT1 activator, interferon-γ (IFN-γ), thus contributing to an inflammatory environment. In normal cells, IFN-γ production is tightly controlled through induction of IFN-γ negative regulators. However, in T-LGLL, IFN-γ signaling lacks this negative feedback mechanism as evidenced by excessive IFN-γ production and decreased levels of suppressors of cytokine signaling 1 (SOCS1), a negative regulator of IFN-γ. Here we characterize the IFN-γ-STAT1 pathway in TL-1 cells, a cell line model of T-LGLL. TL-1 cells exhibited lower IFN-γ receptor protein and mRNA expression compared to an IFN-γ responsive cell line. Furthermore, IFN-γ treatment did not induce JAK2 or STAT1 activation or transcription of IFN-γ-inducible gene targets. However, IFN-β induced p-STAT1 and subsequent STAT1 gene transcription, demonstrating a specific IFN-γ signaling defect in TL-1 cells. We utilized siRNA targeting of STAT1, STAT3, and STAT5b to probe their role in IL-2-mediated IFN-γ regulation. These studies identified STAT5b as a positive regulator of IFN-γ production. We also characterized the relationship between STAT1, STAT3, and STAT5b proteins. Surprisingly, p-STAT1 was positively correlated with STAT3 levels while STAT5b suppressed the activation of both STAT1 and STAT3. Taken together, these results suggest that the dysregulation of the IFN-γ-STAT1 signaling pathway in TL-1 cells likely results from low levels of the IFN-γ receptor. The resulting inability to induce negative feedback regulators explains the observed elevated IL-2 driven IFN-γ production. Future work will elucidate the best way to target this pathway, with the ultimate goal to find a better therapeutic for T-LGLL.

## Introduction

The Janus Kinase (JAK)-Signal Transducers and Activators of Transcription (STAT) pathway is commonly dysregulated in cancers, leading to an upregulation of pro-survival pathways and inflammatory cytokine secretion, including interferon-γ (IFN-γ). IFN-γ, a type II interferon [1], is associated with worse symptomology and disease progression in multiple diseases when produced in excess [2, 3]. IFN-γ directly binds to the IFN-γ receptor (IFNGR), leading to phosphorylation of JAK1, JAK2, and the IFNGR [1, 4–6]. This promotes recruitment and docking of STAT1, allowing activation of STAT1 through phosphorylation of tyrosine residue 701 (p-STAT1) [7]. p-STAT1 then forms a homodimer and moves into the nucleus to transcribe genes with gamma interferon activation site (GAS) elements including IRF-1 and suppressors of cytokine signaling 1 (SOCS1) [4, 7]. SOCS1, a negative regulator of IFN-γ signaling, binds the IFNGR and JAK2 to prevent further activation of the pathway [7]. SOCS1 also participates in cross talk with other pathways, including IL-2-mediated signaling, reducing transcription of IFN-γ [8]. Thus, in healthy cells, IFN-γ signaling is tightly controlled through transcription of negative regulators.

The IFNGR is composed of two subunits, IFNGR1 and IFNGR2, with both subunits required for IFN-γ signaling. IFNGR1 directly interacts with the IFN-γ ligand while IFNGR2 is necessary for signal transduction [1]. Although most cell types moderately and constitutively express IFNGR1, IFNGR2 is responsive to external stimuli and is critically important for downstream IFN-γ-mediated signaling [4, 9]. Higher IFNGR2 expression promotes faster STAT1 phosphorylation and subsequent IRF-1 transcription [9]. Interestingly, IFNGR1 and IFNGR2 levels can be reduced, with profound impacts on downstream signaling. Although the IFNGR is constitutively expressed on most cell types [1, 4], healthy IFN-γ producing cells, such as T helper cells 1 (Th1 cells) or T cell receptor (TCR) engaged T cells, exhibit decreased receptor expression during critical times to prevent apoptosis or induction of negative regulators [9–13]. In contrast to this well-controlled process, IFNGR downregulation can occur in diseased cells [14–18], rendering the cells less responsive or completely unresponsive to IFN-γ signaling. This is particularly problematic as decreased responsiveness would prevent IFN-γ-induced apoptosis of malignant cells. In fact, elegant murine models demonstrate that IFNGR1-/- mice exhibit more rapid ovarian tumor progression [19] and the reduction in IFNGR has been proposed as a mechanism for evading tumor surveillance [16].

In T cell large granular lymphocyte leukemia (T-LGLL), a rare chronic leukemia of CD8+ T cells [20, 21], there is substantial evidence for dysregulation of IFN-γ-mediated signaling. T-LGLL patient serum has significantly elevated IFN-γ compared to healthy controls [22] and T-LGLL patient peripheral blood mononuclear cells (PBMCs) exhibit constitutively activated STAT1 [23], suggesting hyperactivation of the IFN-γ signaling pathway. However, SOCS1 [22] and SOCS3 [24] gene transcripts are significantly reduced in T-LGLL PBMCs, demonstrating a lack of negative regulation. Taken together, we hypothesized that the IFN-γ-STAT1 pathway is dysregulated in T-LGLL.

Here we further characterize the IFN-γ-STAT1 signaling pathway from IFN-γ-induced activation events through the regulation of IFN-γ production in TL-1 cells, a cell line model of T cell large granular lymphocyte leukemia. TL-1 cells, like normal T cells, require IL-2 for proliferation. As IL-2 also drives IFN-γ production [25, 26], we investigated the regulation of IFN-γ production in the context of IL-2 supplementation. Using genetic and pharmacological manipulation of the pathway, we found that TL-1 cells exhibit reduced IFNGR levels compared to Jurkat T cells, an IFN-γ responsive cell line, and were unresponsive to IFN-γ. Moreover, in IL-2-induced signaling, we correlated STAT1 activation with STAT3 protein levels and identified STAT5b as a positive regulator of IFN-γ production and inhibitor of STAT1 in TL-1 cells. Taken together, we propose a novel mechanism that prevents induction of IFN-γ-signaling negative regulators in TL-1 cells. Our findings may have therapeutic implications for T-LGLL based on characterization of the inter-relationship between STAT activation.

## Material and methods

### Reagents

Human IFN-γ1b, premium grade (Cat #130-096-484), human CD119-APC flow cytometry antibody (clone: REA161) (Cat #130-099-920), human IFN-β1a, research grade (Cat #130-107-888), and IL-2 (Cat #130-097-743) were purchased from Miltenyi Biotec. PE anti-human IFN-γ R β chain [2HUB-159] flow cytometry antibody (Cat # 308504) and cell staining buffer (Cat #308504) were purchased from Biolegend. DAPI Solution (Cat #564907) was purchased from BD Pharmigen. OneComp eBeads (Cat #01-1111-42) were purchased from eBiosciences. Radioimmunoprecipitation assay buffer (RIPA) (Cat #R0278) and protease and phosphatase inhibitor cocktails (Cat #P8340, Cat #P5726) were purchased from Sigma Aldrich. FBS (Cat #97068–085) was purchased from Seradigm. Clarity enhanced chemiluminescence (ECL) reagent (Cat #170–5061) and PVDF membrane and filter paper (Cat #170–4274), were purchased from BioRad. RPMI 1640 (Cat #10-00-CV), Pierce bicinchoninic acid (BCA) protein assay kit (Cat #PI23225), and SuperSignal West Femto Maximum Sensitivity Substrate (Cat# 34096) were purchased from ThermoFisher Scientific. Polyacrylamide gels (4–12%; Cat #NW04125BOX) were purchased from Life Technologies. The 3-(4,5-dimethylthiazol-2-yl)-5-(3-carboxymethozyphenyl)-2-(4-sulfonphenyl)-2H-tetrazolium (MTS) Cell Proliferation Colorimetric Assay Kit was purchased from BioVision (Cat #K300-2500). ON-TARGETplus Pooled Human STAT1 siRNA, ON-TARGETplus Pooled Human STAT3 siRNA, and ON-TARGETplus Pooled Human STAT5b siRNA, as well as ON-TARGETplus Control Non-Targeting Pool siRNA (Cat #D-001810-01-05) were purchased from Dharmacon. Ruxolitinib (Cat #S1378) was purchased from Selleckchem.

### Cell culture

Media for all experiments was RPMI 1640 supplemented with 10% FBS. The TL-1 cell line, a model of T-LGLL [27] and the Jurkat T leukemia cell line [28] were used. TL-1 cell medium was supplemented with IL-2 at 200 U/mL and all cells were maintained at 37°C, 5% CO_2_. Cells were plated at a density of 1 million cells/mL, unless indicated. For all IFN-γ treatment and IFNGR experiments, TL-1 cells were IL-2 deprived for 10 h prior to starting the experiment. Ruxolitinib was dissolved in DMSO. For cytokine signaling experiments, cells were treated with 10 ng/mL of human IFN-γ or 5000 U/mL of human IFN-β dissolved in ultra pure DNAse and RNAase free water.

### siRNA-mediated knockdowns

All siRNA knockdown experiments utilized the Invitrogen Neon Transfection System 100 μL Kit (Cat #MPK10096). TL-1 cells were plated at 2.5 million cells/mL and treated with 100 nM STAT1, 50 nM of STAT3, 100 nM of STAT5b, or dose matched scrambled siRNA for 48 h. After 48 h, protein was harvested from a subset of TL-1 cells from each condition to assess knockdown status. The MTS Cell Proliferation Colorimetric Assay Kit was used to assess cell viability after 48 h of siRNA treatment. The reaction was incubated at 37°C, 5% CO_2_ for 1 h, and formazan product was detected on a plate reader at 493 nm (Cytation3 Imaging Reader). Data were normalized to the scrambled control treatment. All conditions were done in quadruplicate. Protein and RNA were harvested from the remaining TL-1 cells from each condition. Conditioned media was harvested for cytokine analysis by Luminex (Millipore Sigma, Cat #HCYTOMAG-60K-06). Jurkat T cells were plated at 0.5 million cells/mL and were treated with 200 nM STAT5b siRNA or 200 nM scrambled siRNA for 48 h before protein and mRNA were harvested.

### RNA extraction and quantitative PCR

For IFN-γ and IFN-β-induced signaling experiments, TL-1 and Jurkat T cells were treated with 10 ng/mL of IFN-γ or 5000 U/mL IFN-β or water for 6 h before RNA harvest. For STAT knockdown experiments, TL-1 and Jurkat T cells were treated as described in the siRNA-mediated knockdown section and then RNA was extracted 48 h after siRNA transfection. For all experiments, cells were harvested and lysed with Invitrogen TRIzol Reagent (Cat #15596018) and stored at -80°C. RNA was isolated using Zymo Research Direct-zol MiniPrep kit (Cat #R2050) and quantified using Invitrogen Qubit RNA Broad Range Assay kit (Cat #Q10210) and Invitrogen Qubit 2.0 Fluorometer (Cat # Q32866). Clontech RNA to cDNA EcoDry Premix (Double Primed) (Cat #639548) was used to reverse transcribe the extracted RNA. BioRad iTaq Universal SYBR Green Supermix (Cat #1725121) and PrimePCR SYBR Green Assay primers were used for all qPCR reactions: STAT1 (Cat #10025636, qHsaCED0043612), IFNGR1 (Cat #10025636, qHsaCID0013339), IFNGR2 (Cat #10025636, qHsaCID0008956), IRF-1 (Cat #10025636, qHsaCED0044080), SOCS1 (Cat #10025636, qHsaCED0002534), IFNG (Cat #10025636, qHsaCID0017614), UBC (Cat #10025636, qHsaCED0023867). Each sample was loaded in triplicate for each primer and each condition had three independent biological replicates. Cycle number and annealing temperature were followed according to the Prime PCR protocol. Results were normalized to vehicle or scrambled control.

### Western blot

Treated cells were washed with PBS then lysed in RIPA buffer (Cat #R0278, Sigma) with protease (Cat #P8340, Sigma) and phosphatase inhibitors (Cat #P5726, Sigma). Protein content was quantified using the BCA assay. Proteins were electrophoresed on a 4–12% polyacrylamide gel and then transferred to a PVDF membrane, blocked with non-fat dried milk or BSA, and incubated with primary antibody at overnight at 4°C according to manufacturer recommendations. Cell Signaling Technology primary antibodies used in these studies were: STAT1 (Cat #9175), Phospho-STAT1 (Y701) (Cat #7649), STAT3 (Cat #9139), Phospho-STAT3 (Tyr 705) XP (Cat #9145), STAT5 (Cat #9363), Phospho-STAT5 (Tyr694) (Cat #9351), JAK2 XP (Cat #3230), Phospho-JAK2 (Tyr1007) (Cat #4406), and β-actin (Cat #3700). After the membrane was washed, it was incubated with secondary antibody (Cell Signaling Technology, anti-rabbit IgG-HRP linked #7074 or anti-mouse IgG-HRP linked #7076) for 1 h then treated with ECL or Femto substrate. Images were captured with a BioRad ChemiDoc MP instrument and analyzed using Image Lab software (BioRad).

### Cytokine analysis

After 48 h treatment with siRNA, aliquots of the conditioned media were collected and stored at -80°C prior to analysis by the UVA Flow Cytometry Core using the Luminex MAGPIX bead-based multiplex analyzer. All conditions were done in triplicate.

### Flow cytometry

TL-1 and Jurkat cells were treated with Human Fc Receptor Binding Inhibitor Purified (Cat #E05904-1648) from eBiosciences and antibodies according to manufacturer protocols. Cells were analyzed using the BD Biosciences LSRFortessa. Appropriate fluorescence minus one (FMO) and compensation controls were used. Living cells were gated based on DAPI staining followed by singlets and green fluorescent protein (GFP) status. TL-1 cells are GFP+ and Jurkat T cells are GFP-, requiring compensation for potential spectral overlap. Median fluorescent intensity of the IFNGR1 and IFNGR2 was determined and compared. Results were analyzed using FCS Express 9 (De Novo Software). All conditions had three biological replicates.

### Statistical analysis

Data were analyzed using GraphPad Prism software version 7. An unpaired 2-tailed Student’s t-test was utilized and P-values of <0.05 were considered significant. For experiments that normalized the control to 1, a Student’s t-test was performed to a hypothetical value of 1.

## Results

### TL-1 cells exhibit decreased IFNGR compared to Jurkat T cells

**Fig 1:**
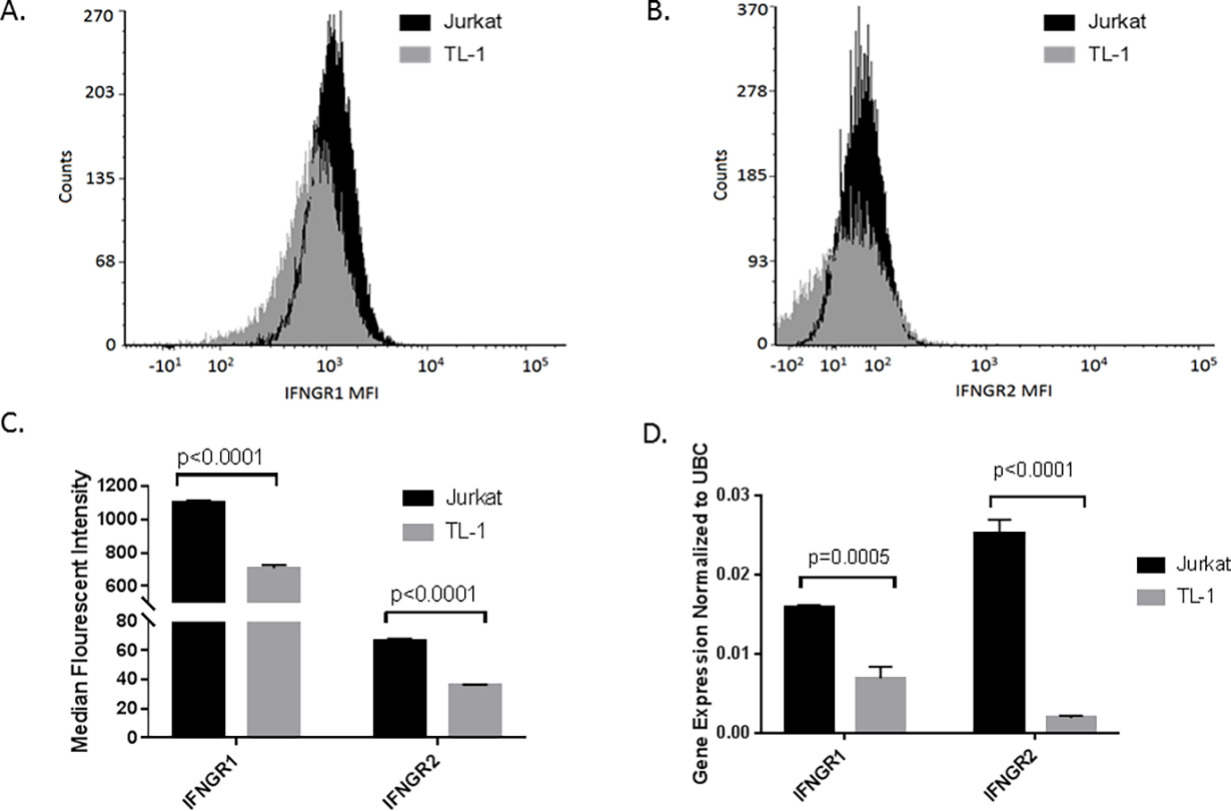
TL-1 cells exhibit lower expression of the IFNGR1 and IFNGR2 compared to Jurkat T cells. The surface expression of the IFNGR1 and IFNGR2 was determined using flow cytometry in the TL-1 cell line compared to Jurkat T cells, an IFN-γ responsive cell line. Representative flow cytometry histograms of (A) IFNGR1 and (B) IFNGR2 and (C) median fluorescence intensity (MFI) of IFNGR1 and IFNGR2 in TL-1 and Jurkat T cells are shown. (D) IFNGR1 and IFNGR2 transcripts were quantified using qPCR. Results were normalized to ubiquitin C (UBC), a housekeeping gene. Student’s T test was used to determine significance of TL-1 cells compared to Jurkat T cells. All data are presented as mean +/- Stdev (n = 3 biological replicates).

Because both IFNGR1 and IFNGR2 are necessary for IFN-γ signaling, we chose to quantify IFNGR1 (**Fig 1A**) and IFNGR2 (**Fig 1B**) cell surface expression in TL-1 cells using flow cytometry. We utilized Jurkat T cells as a positive control cell line known to express IFNGR1 and IFNGR2. Based on median fluorescent intensity, TL-1 cells exhibited 36% (p<0.0001) and 46% (p<0.0001) less IFNGR1 and IFNGR2, respectively, compared to Jurkat T cells (**Fig 1C**). Due to the very low median fluorescent intensity of IFNGR2 in TL-1, we next measured IFNGR1 and IFNGR2 transcript levels to account for potential background. IFNGR1 and IFNGR2 transcripts were 57% (p = 0.0005) and 92% (p<0.0001) lower in TL-1 cells compared to Jurkat T cells (**Fig 1D**), further demonstrating the reduction in IFNGR. Thus, we hypothesized that TL-1 cells would be less responsive or completely unresponsive to IFN-γ treatment compared to Jurkat T cells as a result of lower levels of the IFNGR1 and IFNGR2.

### JAK2 is not phosphorylated in response to IFN-γ treatment in TL-1 cells

**Fig 2:**
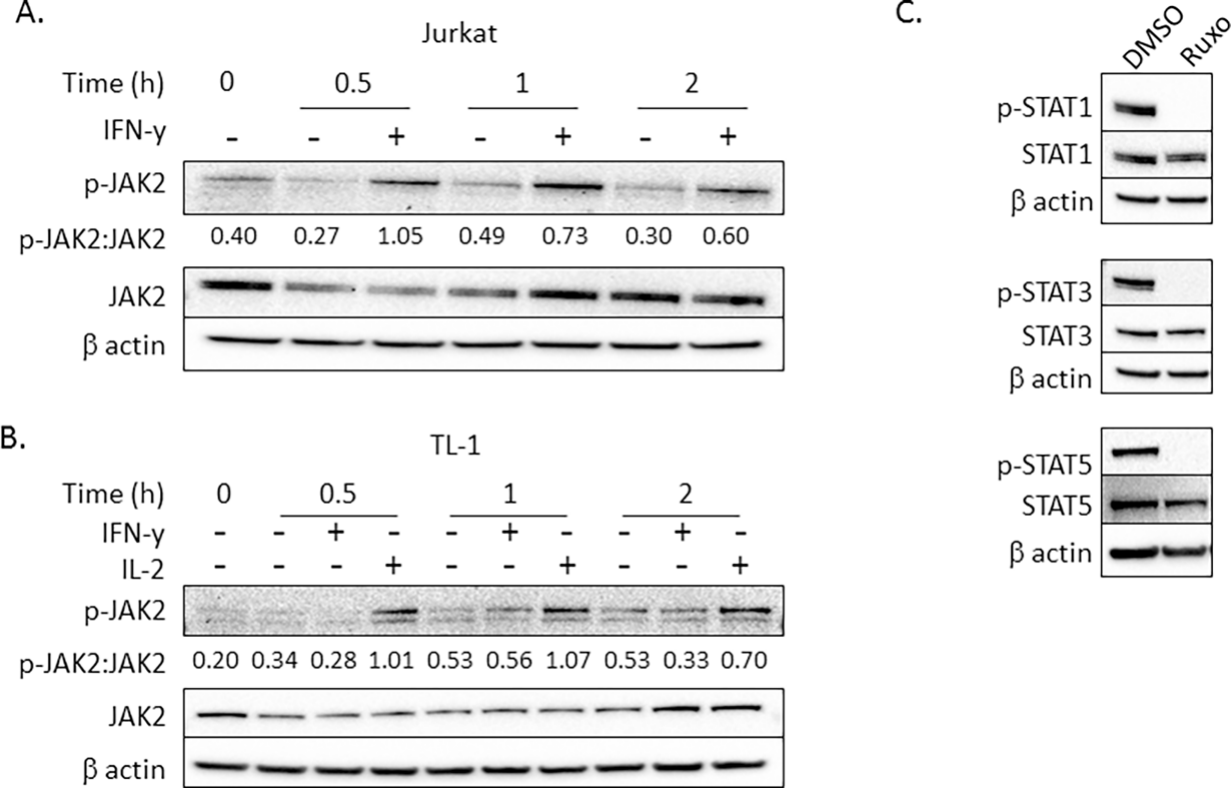
JAK2 is unresponsive to IFN-γ in TL-1 cells. Jurkat T cells (A) or TL-1 cells (B) were treated with 10 ng/mL IFN-γ or water (vehicle control) for the indicated time. TL-1 cells were also treated with IL-2 as a positive control for induction of p-JAK2. p-JAK2, total JAK2, and β actin were measured using western blot. The ratio of p-JAK2:JAK2 is shown for each condition. (C) TL-1 cells were pre-treated with 5 μM ruxolitinib (Ruxo) or DMSO for 2 h prior to the addition of IL-2. Protein lysates were created 1 h after the addition of IL-2. Western blots were probed for p-STAT1, total STAT1, and β actin. p-STAT3, total STAT3, p-STAT5 and total STAT5 were used as controls for the functionality of the JAK inhibitors.

**Fig 3:**
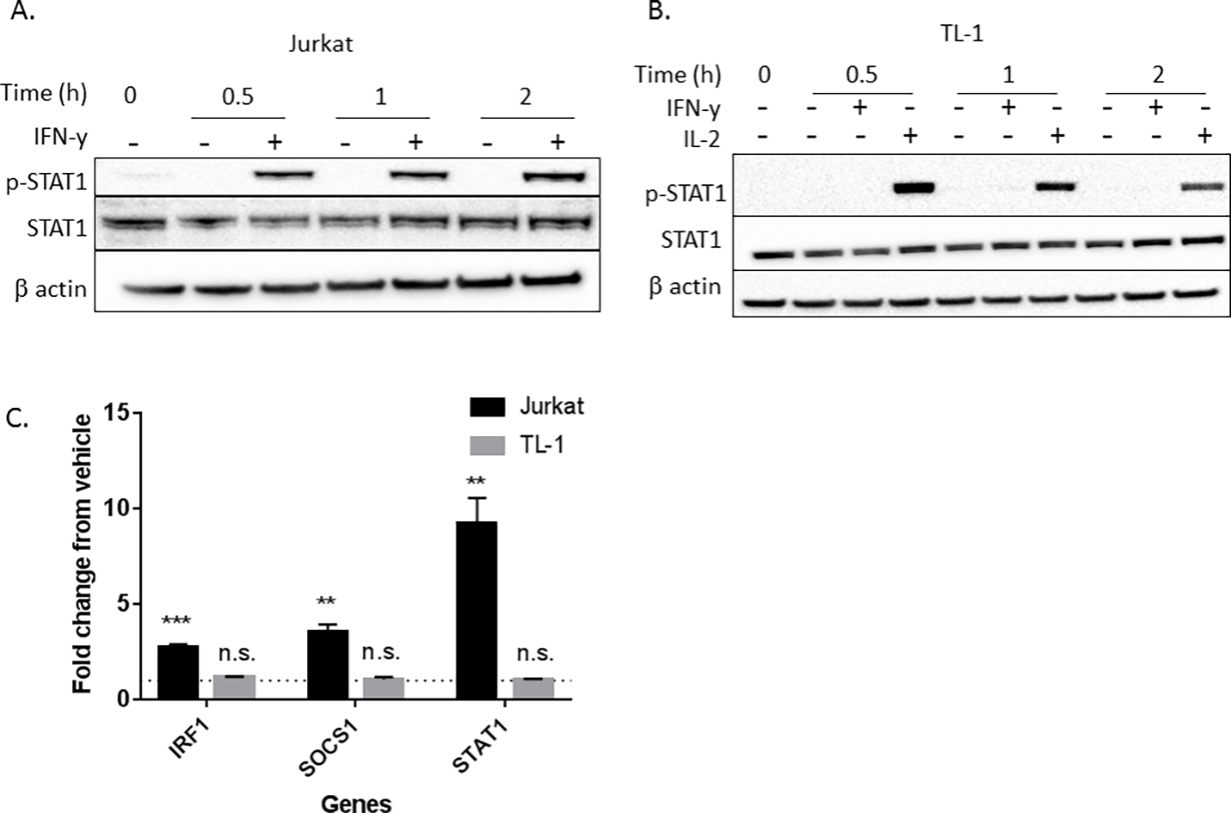
IFN-γ does not activate STAT1 or induce transcription of IFN-γ-regulated genes in TL-1 cells. Jurkat T cells (A) or TL-1 cells (B) were treated with 10 ng/mL IFN-γ or water (vehicle control) for the indicated time. TL-1 cells were also treated with 200 U/mL IL-2 as a positive control for induction of p-STAT1. p-STAT1, total STAT1, and β actin were measured using western blot. (C) Jurkat or TL-1 cells were treated with 10 ng/mL IFN-γ or water for 6 h prior to RNA extraction. Induction of IRF-1, SOCS1, and STAT1 transcripts was quantified using qPCR. Results were normalized to UBC and then to the water control to demonstrate fold change. Student’s T test was used to determine significance compared to vehicle control. * = p<0.05, ** = p<0.01, *** = p<0.005, **** = p<0.001. Data are presented as mean +/- Stdev (n = 3 biological replicates).

**Fig 4:**
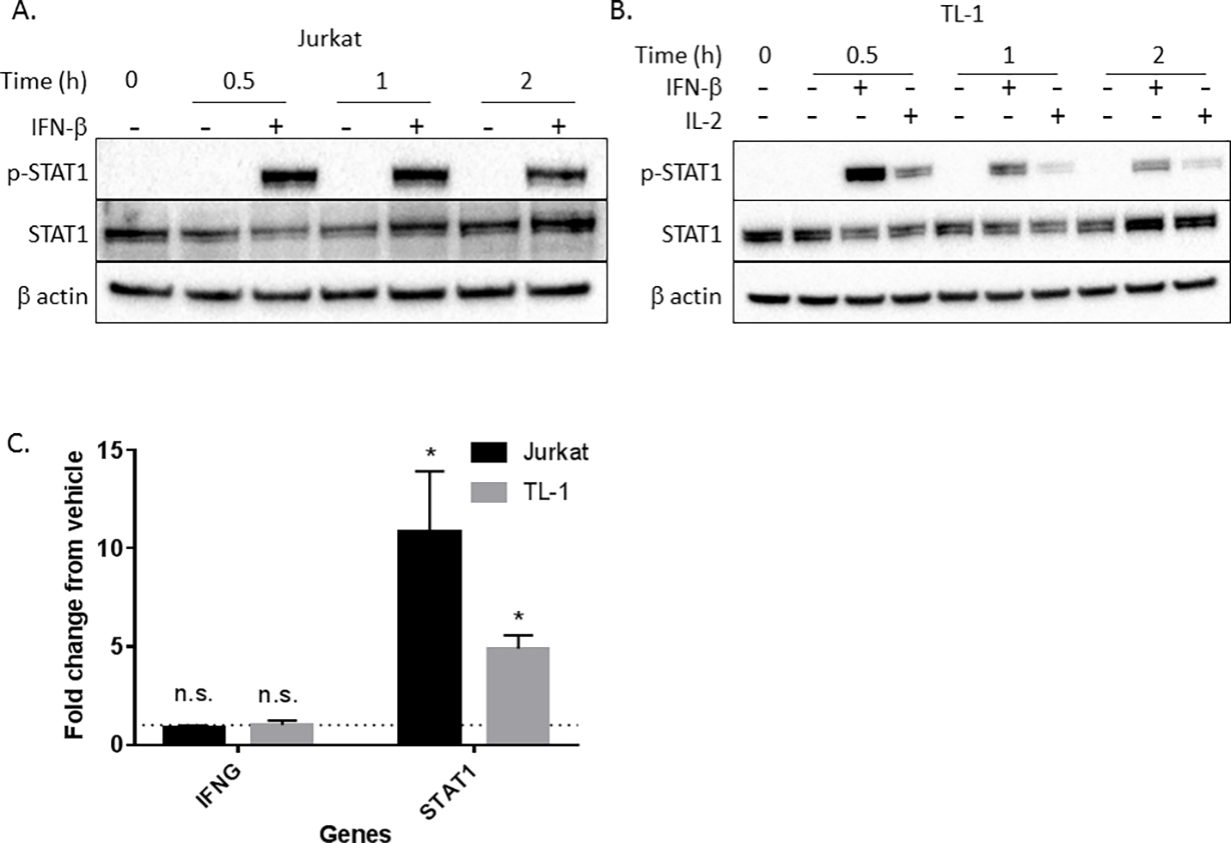
TL-1 cells are responsive to IFN-β, supporting a type II interferon-specific signaling defect. Jurkat T cells (A) or IL-2-starved TL-1 cells (B) were treated with 5000 U/mL IFN-β or water (vehicle control) for the indicated time. TL-1 cells were also treated with 200 U/mL IL-2 as a positive control for induction of p-STAT1. p-STAT1, total STAT1, and β actin were measured using western blot. (C) Jurkat or TL-1 cells were treated with 5000 U/mL IFN-β or water for 6 h prior to RNA extraction. Induction of IFN-γ and STAT1 transcripts was quantified using qPCR. Results were normalized to UBC (a housekeeping gene) and then to the water control to demonstrate fold change. Student’s T test was used to determine significance compared to vehicle control. * = p<0.05, ** = p<0.01, *** = p<0.005, **** = p<0.001. Data are presented as mean +/- Stdev (n = 3 biological replicates).

To assess whether the lower levels of IFNGR affect downstream kinase activation, we measured JAK2 phosphorylation following IFN-γ treatment in TL-1 cells compared to Jurkat T cells, our positive control. As expected, IFN-γ activated JAK2 within 0.5 h with effects lasting at least 2 h in Jurkat T cells (**Fig 2A**). However, in TL-1 cells p-JAK2 was not induced with IFN-γ (**Fig 2B**), demonstrating an IFN-γ unresponsive state in TL-1 cells. IL-2 was selected as a positive control cytokine for induction of JAK2, STAT1, STAT3, and STAT5 in TL-1 cells (**Figs 2B, 3B and 4B**). We have previously demonstrated that the TL-1 cell line is responsive to IL-2 [29, 30]. In the present manuscript, we demonstrate that the cytokine unresponsiveness appeared to be unique for IFN-γ and not an inherent defect in JAK2 activation as p-JAK2 was induced in TL-1 cells in response to IL-2 (**Fig 2B**). Since JAK2 is required for IFN-γ-induced STAT1 activation [4, 6], we determined whether the JAK2-STAT1 interaction is intact in TL-1 cells. We utilized ruxolitinib, a JAK1 and JAK2 specific inhibitor, and measured IL-2-induced STAT1 phosphorylation. JAK1/JAK2 activity is required for activation of STAT1 as p-STAT1 was completely ablated with ruxolitinib (**Fig 2C**). p-STAT3 and p-STAT5 were also reduced (**Fig 2C**). Therefore, JAK2 is phosphorylated and activates downstream STATs in response to IL-2 treatment, but not IFN-γ, in TL-1 cells.

### IFN-γ does not activate STAT1 or induce transcription of IFN-γ-inducible gene targets in TL-1 cells

As p-JAK2 was not induced by IFN-γ in TL-1 cells, we hypothesized that STAT1 would not be activated as a result of IFN-γ treatment. Following IFN-γ treatment, Jurkat T cells exhibited robust STAT1 activation at 0.5 h, with activation lasting at least 2 h (**Fig 3A**). On the contrary, p-STAT1 was undetectable in TL-1 cells at any time point following IFN-γ treatment but was detectable following IL-2 treatment, again demonstrating an IFN-γ specific defect (**Fig 3B**). Neither STAT3 nor STAT5 activation was detected following IFN-γ treatment, while IL-2 robustly induced STAT3 and STAT5 phosphorylation (**S1 Fig**), further demonstrating a specific lack of IFN-γ signaling. Given the inability to detect p-STAT1 in IFN-γ treated TL-1 cells, we next assessed whether STAT1-mediated-IFN-γ-inducible gene targets would be upregulated in response to IFN-γ treatment. TL-1 and Jurkat T cells were treated with IFN-γ for 6 h and fold change in transcript levels of target genes was measured and compared. IRF1, SOCS1, and STAT1 mRNA levels were increased by 2.7- (p<0.005), 3.5- (p<0.01), and 9.2-fold (p<0.01), respectively, in Jurkat T cells treated with IFN-γ compared to the vehicle control (**Fig 3C**). In contrast, IFN-γ treatment of TL-1 cells did not induce transcription of IRF1, SOCS1, or STAT1 (**Fig 3C**). These findings further demonstrate the IFN-γ unresponsive state of TL-1 cells.

### TL-1 cells are responsive to IFN-β, suggesting a specific IFN-γ-mediated signaling defect independent of type I interferon signaling

There is mounting support for crosstalk between type I and type II interferon pathways. There are several different type I interferons including IFN-α, IFN-β, IFN-ω, and IFN-κ, while only IFN-γ comprises the type II interferons [1]. Unlike IFN-γ, which signals through its unique receptor, all type I interferons signal through the IFN-α receptor 1 (IFNAR1) and IFN-α receptor 2 (IFNAR2) [1]. Although signaling occurs through different receptors, lack of IFNAR can lead to a diminished IFN-γ signaling pathway, implicating IFNAR in regulation of IFN-γ responsiveness [31]. To address whether the lack of STAT1 induction in TL-1 cells is specific to IFN-γ or a result of diminished IFNAR-mediated signaling, we assessed the ability of TL-1 cells to respond to IFN-β. TL-1 cells were treated with 5000 U/mL of IFN-β for up to 2 h before induction of p-STAT1 was assessed, with Jurkat T cells serving as our positive control for IFN-β responsiveness (**Fig 4A**). IFN-β robustly induced p-STAT1 in TL-1 cells within 0.5 h (**Fig 4B**). Treatment of Jurkat T cells and TL-1 cells with IFN-β led to a significant increase in STAT1 gene transcription (p<0.05) but not IFN-γ (**Fig 4C**). Therefore, IFNAR signaling is intact and functional in TL-1 cells despite the lack of IFN-γ responsiveness.

### STAT5b promotes transcription of IFN-γ

**Fig 5:**
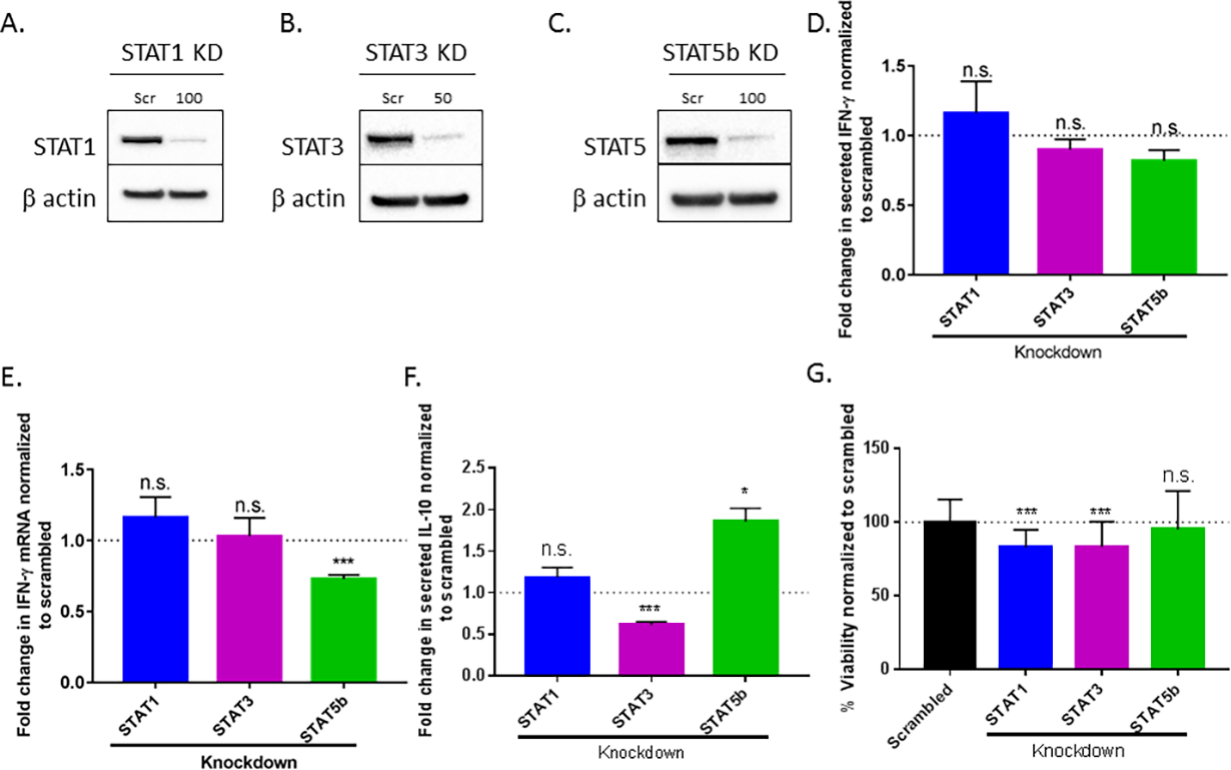
STAT5b is required for maximal production of IL-2-induced IFN-γ mRNA in TL-1 cells. STAT1 (A), STAT3 (B), or STAT5b (C) were knocked down using siRNA in TL-1 cells supplemented with IL-2. Protein lysates and RNA were harvested as well as conditioned media collected 48 h after siRNA transfection. Representative western blots of the knockdowns are shown. (D) Supernatant was analyzed to determine changes in secreted IFN-γ as a result of each knockdown. (E) The effect of knockdown on IFN-γ transcript levels was determined using qPCR. Results were normalized to the UBC gene and further normalized to the scrambled siRNA control. (F) Changes in IL-10 production were also assessed in each knockdown group using Luminex. (G) Viability following knockdown was measured by MTS assay at 48h. All results were normalized to scrambled siRNA treated cells. Student’s T test was used to determine significance compared to scrambled siRNA. * = p<0.05, ** = p<0.01, *** = p<0.005, **** = p<0.001. Data are presented as mean +/- Stdev (n = 3 biological replicates).

The significantly elevated IFN-γ production by T-LGLL cells coupled with the decreased levels of SOCS1 suggests that there is a lack of negative regulation in T-LGLL [22]. Our results demonstrate that IFN-γ treatment does not induce the IFN-γ negative regulator, SOCS1 (**Fig 3C**). Thus, it is key that we identify IFN-γ transcription factors that may be targeted to reduce the production of IFN-γ and prevent further damage to healthy cells. Since STAT1, STAT3, and STAT5 have been shown to transcribe IFN-γ [32–36] and play a role in T-LGLL pathogenesis [23, 37], we chose to focus on these transcription factors in the context of IL-2 supplementation. STAT1 (**Fig 5A**), STAT3 (**Fig 5B**), or STAT5b (**Fig 5C**) siRNA was transfected into TL-1 cells to knockdown their respective protein levels. STAT1 and STAT3 protein knockdown did not affect IFN-γ protein (**Fig 5D**) or transcript levels (**Fig 5E**). However, STAT5b knockdown resulted in a 19% decrease in secreted IFN-γ protein (p = 0.055) (**Fig 5D**) and 28% decrease in IFN-γ transcript (p<0.005) (**Fig 5E**) 48 h after STAT5b siRNA treatment, implicating STAT5b in the regulation of IFN-γ. STAT5b knockdown also reduced IFN-γ mRNA content by 34% in Jurkat T cells (**S2 Fig**), demonstrating that this relationship is conserved across cell lines.

Because IFN-γ and IL-10 classically work in opposition [38], we next investigated the effects of the STAT knockdowns on IL-10 production in TL-1 cells. As expected, IFN-γ and IL-10 production were inversely related with a nearly 2-fold increase in IL-10 in STAT5b knockdown cells (p<0.05) (**Fig 5F**) while IFN-γ was decreased (**Fig 5E**). STAT3 knockdown cells exhibited a 39% decrease in IL-10 (p<0.005) (**Fig 5F**), matching the established role of STAT3 in regulating IL-10 production [39], while STAT1 knockdown cells showed no significant change in IL-10 production. These changes in cytokine production were not associated with any dramatic differences in cell viability (**Fig 5G**). As STAT5b alters cytokine production, we investigated whether STAT5b knockdown changed IFNGR1 or IFNGR2 mRNA levels. IFNGR1 was significantly (p<0.05) reduced while IFNGR2 was not altered in TL-1 cells following STAT5b knockdown (**S3 Fig**). Taken together, STAT5b is required for optimal production of IFN-γ and IFNGR1 mRNA in TL-1 cells.

### STAT5b suppresses STAT1 and STAT3 activation in TL-1 cells

**Fig 6:**
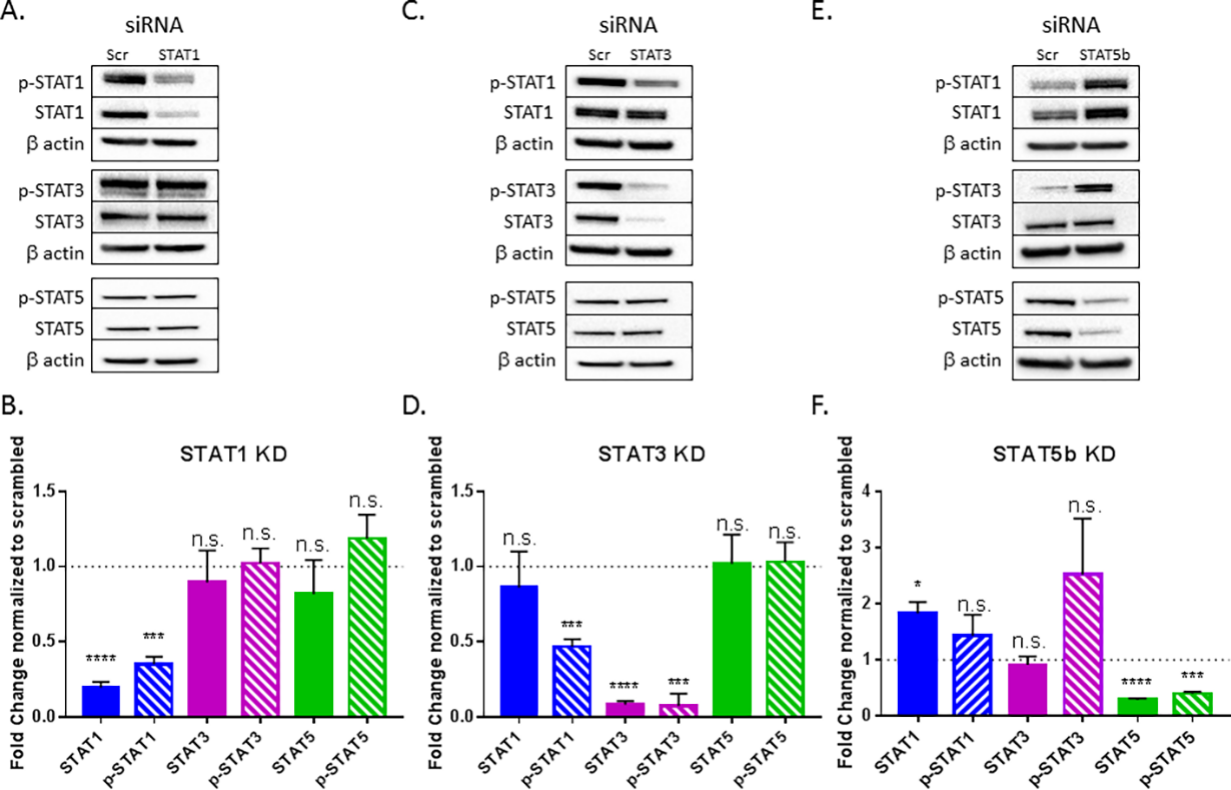
STAT5b inhibits STAT1 and STAT3 activation in TL-1 cells. STAT1 (A, B), STAT3 (C, D), and STAT5b (E, F) were knocked down using siRNA in IL-2 supplemented TL-1 cells. Protein lysates were harvested 48 h after siRNA transfection. Each blot was probed for p-STAT1, STAT1, p-STAT3, STAT3, p-STAT5, STAT5, and β actin. p-STAT proteins were normalized to their respective total STAT proteins. STAT1, STAT3, and STAT5 were normalized to β actin. Representative western blots are shown (A, C, E) as well as quantification of experimental replicates (B, D, F). Student’s T test was used to determine significance compared to scrambled siRNA. * = p<0.05, ** = p<0.01, *** = p<0.005, **** = p<0.001. Data are presented as mean +/- Stdev (n = 3 biological replicates).

Given the role of STAT1, STAT3, and STAT5 in T-LGLL pathogenesis, we investigated the relationship between STAT1, STAT3, and STAT5 activation. This would allow us to better understand how inhibiting the STATs individually, in an effort to target cytokine production and induce apoptosis, would impact STAT signaling in T-LGLL. We next determined whether selected STAT knockdowns produced compensatory changes in other STATs. Knockdown of STAT1 did not impact phosphorylated or total protein levels of STAT3 or STAT5 (**Fig 6A and 6B).** STAT3 knockdown led to a significant reduction in p-STAT1 (p<0.005), with phosphorylated and total protein level of STAT5 unchanged (**Fig 6C and 6D**). However, when STAT5b was knocked down, there was a significant increase in total STAT1 (p<0.05) as well as a notable increase in p-STAT1 and p-STAT3 (**Fig 6E and 6F**), demonstrating a suppressive effect of STAT5b. Taken together, our findings suggest STAT5b inhibits STAT1 while STAT3 promotes STAT1 activation.

## Discussion

Our results suggest that TL-1 cells are nonresponsive to IFN-γ-mediated signaling. Here we demonstrate that the TL-1 cell line model of T-LGLL exhibits minimal protein and mRNA levels of both IFNGR1 and IFNGR2 (**Fig 1**). Moreover, downstream IFN-γ-mediated signaling is deficient, with a lack of induction of p-JAK2 (**Fig 2**), p-STAT1 (**Fig 3**), or IFN-γ-inducible gene targets including SOCS1, IRF1, and STAT1 (**Fig 3**). The low expression of the IFNGR may provide a mechanistic basis for the known lack of IFN-γ-induced negative regulation yet high levels of IFN-γ production in T-LGLL [22]. The impairment of SOCS1 induction would prevent the negative feedback regulation of IL-2-mediated IFN-γ production, which is observed as elevated IFN-γ levels in primary T-LGLL patient samples and the TL-1 cell line.

For our experiments, we utilized Jurkat T cells as an IFN-γ responsive cell line. However, Jurkat T cells are considered slow responders to IFN-γ due to established lower IFNGR2 levels compared to other IFN-γ responsive cell lines [9, 11]. Thus, our findings of greater observed reduction in IFNGR1 and IFNGR2 in TL-1 cells, relative to Jurkat T cells, are even more remarkable. Such low receptor expression renders TL-1 cells unresponsive to IFN-γ treatment (**Figs 2 and 3**). Decreased expression of IFNGR can be due to receptor internalization, degradation, or suppressed transcription of the receptor subunits [15, 18]. Based on our mRNA data (**Fig 1**), we found that TL-1 cells have fewer IFNGR transcripts, resulting in less IFNGR protein, compared to Jurkat T cells. Moreover, our results suggest that STAT5b may play a role in regulating IFNGR1 mRNA levels but not IFNGR2 mRNA levels (**S3 Fig**).

Furthermore, we demonstrate that the lack of IFN-γ responsiveness is not due to defects in the type I interferon signaling pathway (**Fig 4**). Deficiency in the type I interferon receptors IFNAR1 and IFNAR2 has been shown to dampen IFN-γ signaling [31]. However, we found that TL-1 cells robustly induce p-STAT1 in response to IFN-β treatment (**Fig 4**). These results demonstrate three concepts. First, a deficiency in IFN-β signaling is not responsible for the unresponsiveness of TL-1 cells to IFN-γ. Second, IFN-β signaling and IL-2 signaling (**Figs 2, 3 and 4 and S1 Fig**) are both intact, which supports a specific IFN-γ signaling defect in TL-1 cells. Third, the deficiency in IFN-γ signaling does not impact the ability of TL-1 cells to respond to IFN-β. To the best of our knowledge, this is the first study to evaluate type I interferon STAT signaling in LGL leukemia.

The JAK-STAT pathway is commonly dysregulated in malignancies leading to an increase in inflammatory cytokine production and a decrease in induction of pro-apoptotic pathways [40]. In T-LGLL, STAT1 and STAT3 are constitutively activated [23]. Approximately 40% of T-LGLL patients have activating somatic mutations in *STAT3* [37], and a small percentage of T-LGLL patients have activating *STAT5b* somatic mutations [41]. As STAT1, STAT3, and STAT5b have been identified as important transcription factors in T-LGLL pathogenesis [23, 37, 41, 42], we investigated the role of these transcription factors in IL-2-driven IFN-γ production. Our studies with specific siRNA-mediated targeting of STAT proteins suggest that STAT5b, rather than STAT1 and STAT3, drives IFN-γ production in TL-1 cells under IL-2-stimulated conditions (**Fig 5**). We confirmed that STAT5b regulates IFN-γ levels in Jurkat T cells (**S2 Fig**) and other studies also indicate a role of STAT5 in IFN-γ regulation [32, 33, 35, 36]. Specifically, when supplemented with IL-2, as done with the TL-1 cell line, STAT5 has been shown to drive IFN-γ production [32, 34] and when STAT5 is inhibited pharmacologically, IFN-γ decreases [43].

The IL-2-mediated production of IFN-γ by STAT5b can be explained through the previously observed decreased SOCS1 levels. SOCS1, although classically associated with IFN-γ-mediated signaling, acts on several other cytokine-mediated pathways, including those mediated by IL-2 [8]. SOCS1 associates with IL-2Rβ, leading to an inhibition of IL-2-induced STAT5 activation [8]. Thus, the lack of SOCS1 would allow STAT5b to continue to produce IFN-γ without negative regulation in the presence of IL-2, which is in agreement with our observations. Other transcription factors that are known to regulate IFN-γ in CD8+ T cells, such as T-bet [44, 45], Eomes [45], and STAT4 [35, 45], may also be considered for future studies to more completely define the regulators of IFN-γ production in T-LGLL and assess the effects of SOCS1 deficiency on their regulation.

In additional to the role of STAT5b in promoting IFN-γ production, STAT5b significantly represses STAT1 protein levels and moderately decreases STAT3 activation in TL-1 cells (**Fig 6**). This matches the literature as STAT3 and STAT5 have been documented to have opposing roles in malignancies and development [46, 47], with competitive DNA binding to the same site [39, 48–50], leading to displacement of STAT3 [48]. STAT1 and STAT5 have also been shown to have an inverse relationship. STAT1 gain of function in natural killer cells leads to impaired STAT5 activation [51], suggesting antagonistic regulation between these two STATs.

Contrary to its inverse relationship with STAT5b, p-STAT1 positively correlates with p-STAT3 in the TL-1 cell line (**Fig 6**). Although STAT1 and STAT3 are commonly associated with opposing cellular roles [52–54], STAT3 gene expression can enhance STAT1 activation [55] and STAT3 can form heterodimers with STAT1 [56]. In TL-1 cells, STAT1 and STAT3 are simultaneously activated in response to IL-2 and are dually reduced in response to vitamin D [30], suggesting a cooperative relationship between the two STATs. Moreover, in T-LGLL specifically, STAT1 and STAT3 are both constitutively activated and form heterodimers in patient leukemic cells [23]. The mechanism behind STAT3-mediated STAT1 activation in T-LGLL is currently unclear, although likely explanations include production of activating cytokines or the documented STAT1-STAT3 heterodimer formation in T-LGLL cells [23].

**Fig 7:**
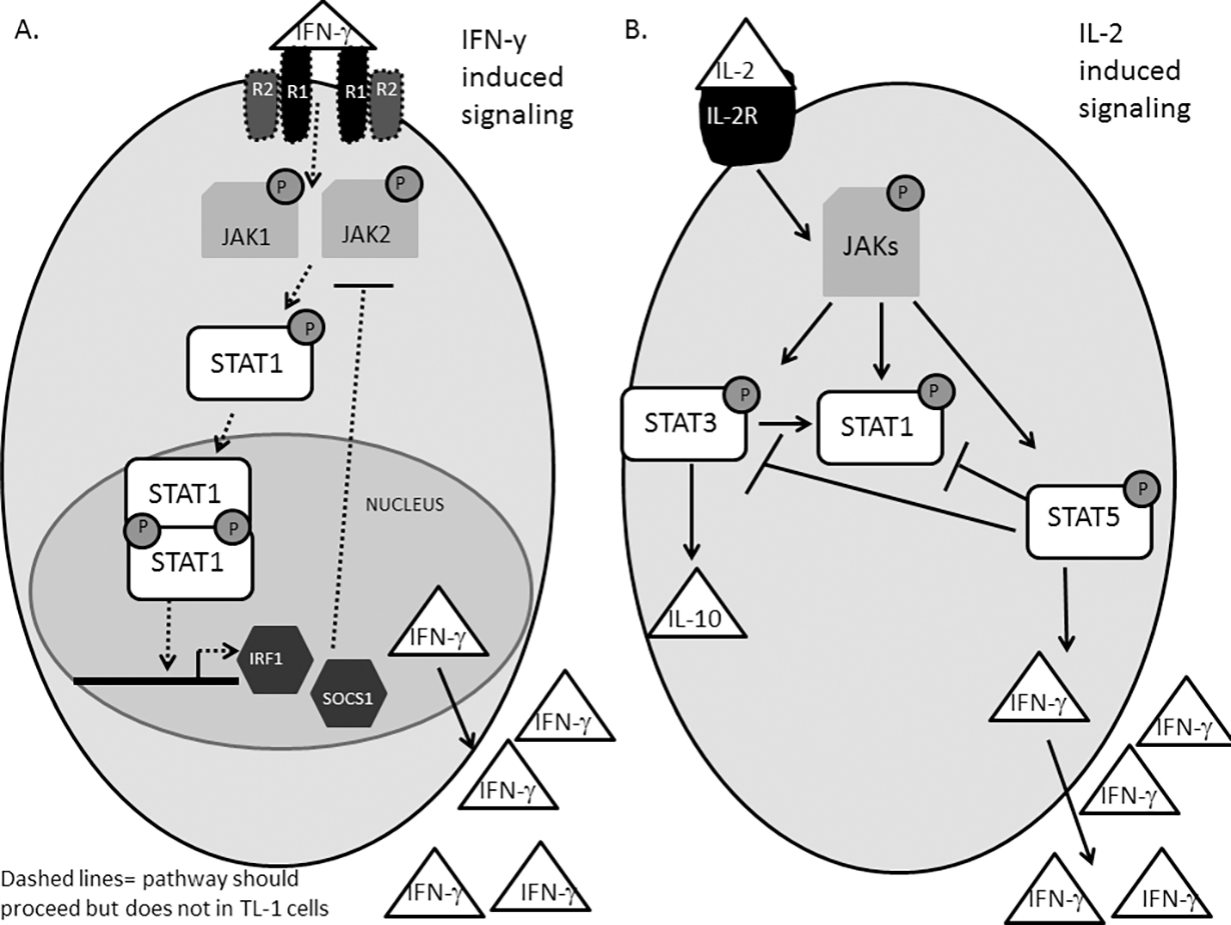
Working model for JAK-STAT signaling pathway in TL-1 cells. (A) Based on our findings, TL-1 cells have a decreased expression of IFNGR1 and IFNGR2, rendering the cells unresponsive to IFN-γ-induced signaling. This allows uncontrolled production of IL-2 induced IFN-**γ** production due to lack of induction of negative feedback regulators. (B) However, TL-1 cells are responsive to IL-2 leading to activation of STAT1, STAT3, and STAT5. STAT1 activation positively correlates with STAT3 while the activation of these proteins is enhanced upon knockdown of STAT5b. STAT5b and STAT3 promote transcription of IFN-γ and IL-10, respectively.

In this current study, we evaluated the IFN-γ signaling pathway and IL-2-induced production of IFN-γ in the TL-1 cell line (**Fig 7**). The main finding is that TL-1 cells are unresponsive to IFN-γ supplementation most likely due to decreased IFNGR expression. Furthermore, the lack of IFN-γ-mediated SOCS1 expression allows IL-2 to drive STAT5b-mediated production of IFN-γ without negative regulation. Although receptor-targeting antibodies are gaining traction in cancer research, our results suggest that IFNGR targeting would be ineffective in inhibiting IFN-γ-induced signaling, given that these cells do not have adequate IFNGR levels. To inhibit excessive IFN-γ production in TL-1 cells, the positive regulators of IFN-γ, including JAKs, STATs, and other cytokines that drive its expression could be targeted. Additionally, the TL-1 cell line offers a novel model for better understanding diseases that exhibit similar unresponsiveness to IFN-γ as a result of decreased IFNGR levels [14–18]. Several studies have demonstrated lower IFNGR levels in primary cells in multiple diseases [14–18]. However the use of primary cells poses limitations for determining long term effects or allowing manipulation of the IFN-γ pathway or related pathways. Through utilization of the TL-1 cell line, potential cross talk with other critical signaling pathways, such as IL-2-mediating JAK-STAT signaling, could be elucidated. Such studies might validate novel therapeutic targets for such IFNGR non-responsive diseases.

## Supporting information

S1 Fig STAT3 and STAT5 phosphorylation is induced by IL-2, but not IFN-γ, in TL-1 cells. IL-2-starved TL-1 cells were treated with 10 ng/mL IFN-γ, 200 U/mL IL-2 (positive control), or water (vehicle control) for the indicated time. p-STAT3 and total STAT3 (A) or p-STAT5 and total STAT5 (B) were measured using western blot. β actin was used as a loading control.
(TIF)

S2 Fig STAT5b regulates IFN-γ mRNA content in Jurkat T cells. STAT5b was knocked down using siRNA in Jurkat T cells. Protein lysates and RNA were harvested 48 h after siRNA transfection. (A) Western blot quantification of STAT5 protein with β-actin as a loading control. (B) The effect of knockdown on IFN-γ transcript levels was determined using qPCR. Results were normalized to UBC, a housekeeping gene, and further normalized to scrambled siRNA.
(TIF)

S3 Fig STAT5b regulates IFNGR1, but not IFNGR2, in TL-1 cells. STAT5b was knocked down using siRNA in TL-1 cells supplemented with IL-2. Protein lysates and RNA were harvested 48 h after siRNA transfection. Representative western blots of the knockdowns can be found in Fig 5C. The effect of knockdown on IFNGR1 and IFNGR2 transcript levels were determined using qPCR. Results were normalized to UBC, a housekeeping gene, and further normalized to the scrambled siRNA control. Student’s T test was used to determine significance compared to scrambled siRNA. * = p<0.05, ** = p<0.01, *** = p<0.005, **** = p<0.001. Data are presented as mean +/- Stdev (n = 3 biological replicates).
(TIF)

## Abbreviations

IFN-γ: interferon gamma
IFN-β: interferon beta
IL: interleukin
JAK: Janus kinase
T-LGL: T cell large granular lymphocyte
T-LGLL: T cell large granular lymphocyte leukemia
MTS: 3-(4,5-dimethylthiazol-2-yl)-5-(3-carboxymethoxyphenyl)-2-(4-sulfophenyl)-2H-tetrazolium
PBMC: peripheral blood mononuclear cell
STAT: signal transducer and activator of transcription
IFNGR: interferon gamma receptor
IRF-1: interferon regulatory factor 1
SOCS: suppressors of cytokine signaling
UBC: ubiquitin C
