# Supplementary figures and images for: Dysregulation of the IFN-γ-STAT1 signaling pathway in a cell line model of large granular lymphocyte leukemia
Source: PLoS One. 2018 Feb 23;13(2):e0193429. doi: 10.1371/journal.pone.0193429 (PMC5825082; doi:10.1371/journal.pone.0193429)

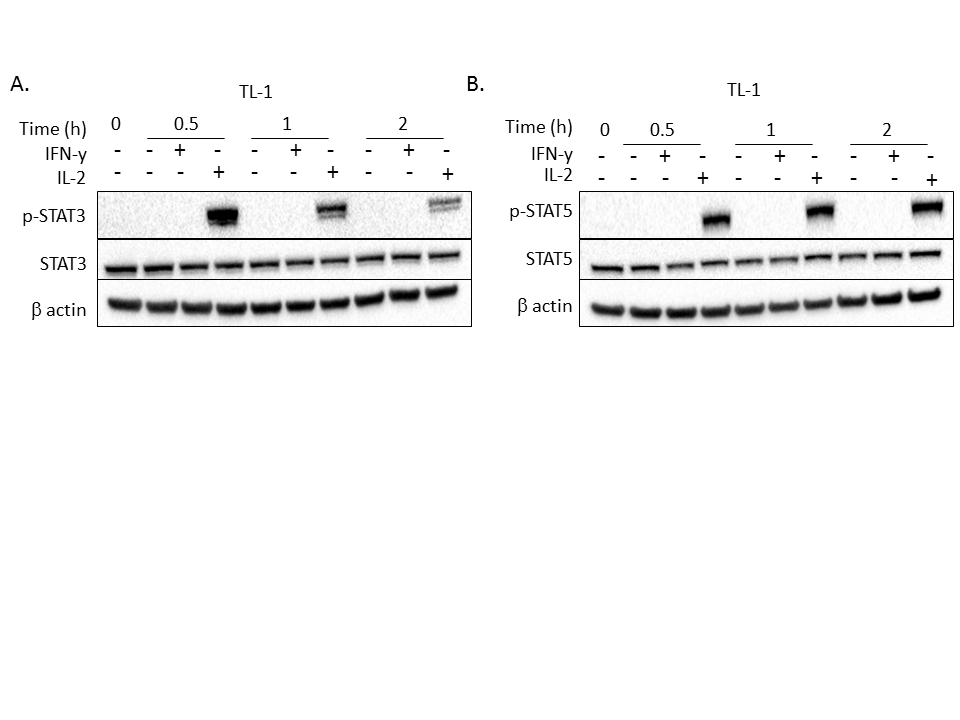

Supplement: S1 Fig — IL-2-starved TL-1 cells were treated with 10 ng/mL IFN-γ, 200 U/mL IL-2 (positive control), or water (vehicle control) for the indicated time. p-STAT3 and total STAT3 (A) or p-STAT5 and total STAT5 (B) were measured using western blot. β actin was used as a loading control. (TIF) [file pone.0193429.s001.tif]

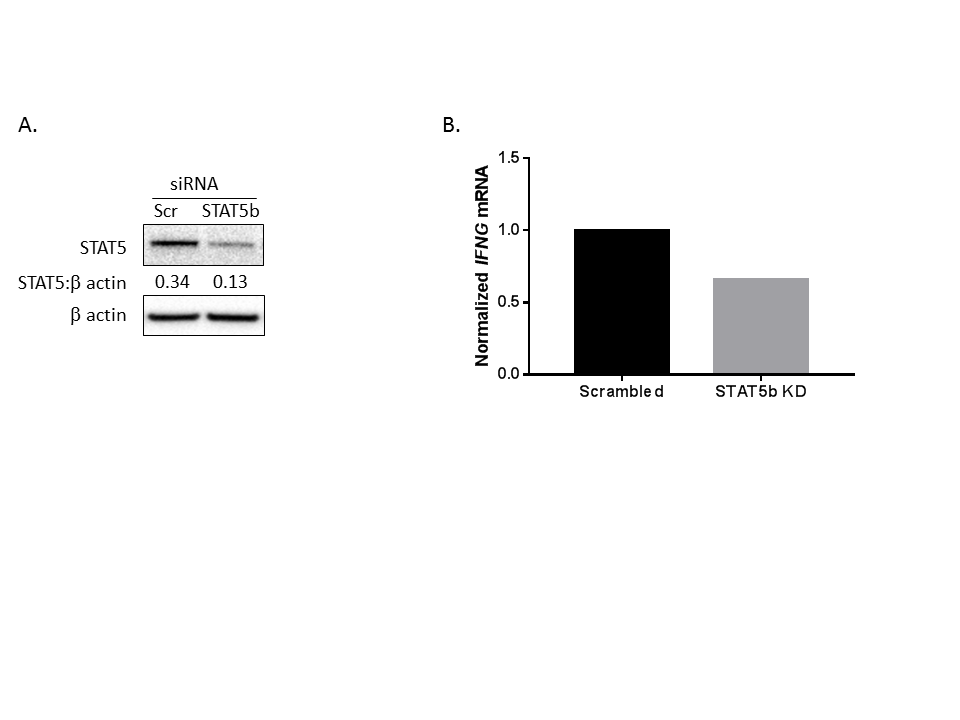

Supplement: S2 Fig — STAT5b was knocked down using siRNA in Jurkat T cells. Protein lysates and RNA were harvested 48 h after siRNA transfection. (A) Western blot quantification of STAT5 protein with β-actin as a loading control. (B) The effect of knockdown on IFN-γ transcript levels was determined using qPCR. Results were normalized to UBC, a housekeeping gene, and further normalized to scrambled siRNA. (TIF) [file pone.0193429.s002.tif]

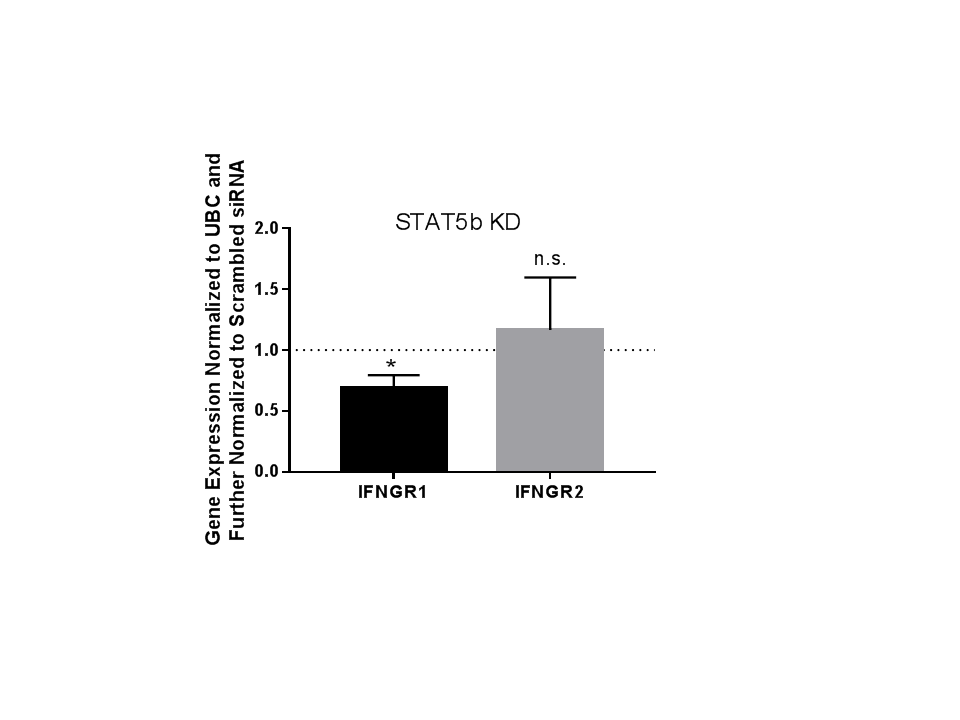

Supplement: S3 Fig — STAT5b was knocked down using siRNA in TL-1 cells supplemented with IL-2. Protein lysates and RNA were harvested 48 h after siRNA transfection. Representative western blots of the knockdowns can be found in Fig 5C. The effect of knockdown on IFNGR1 and IFNGR2 transcript levels were determined using qPCR. Results were normalized to UBC, a housekeeping gene, and further normalized to the scrambled siRNA control. Student’s T test was used to determine significance compared to scrambled siRNA. * = p<0.05, ** = p<0.01, *** = p<0.005, **** = p<0.001. Data are presented as mean +/- Stdev (n = 3 biological replicates). (TIF) [file pone.0193429.s003.tif]
